# Machine learning-based ultrasomics for predicting response to tyrosine kinase inhibitor in combination with anti-PD-1 antibody immunotherapy in hepatocellular carcinoma: a two-center study

**DOI:** 10.3389/fonc.2024.1464735

**Published:** 2024-11-14

**Authors:** Yiwen Hu, Linlin Zhang, Qinghua Qi, Shanshan Ren, Simeng Wang, Lanling Yang, Juan Zhang, Yuanyuan Liu, Xiaoxiao Li, Xiguo Cai, Shaobo Duan, Lianzhong Zhang

**Affiliations:** ^1^ Department of Ultrasound, Henan University People’s Hospital, Henan Provincial People’s Hospital, Zhengzhou University People’s Hospital, Zhengzhou, China; ^2^ Henan Engineering Technology Research Center of Ultrasonic Molecular Imaging and Nanotechnology, Henan Provincial People’s Hospital, Zhengzhou, China; ^3^ Department of Ultrasound, First Affiliated Hospital of Zhengzhou University, Zhengzhou, China; ^4^ Henan Rehabilitation Clinical Medical Research Center, Henan Provincial People’s Hospital, Zhengzhou, China; ^5^ Henan Key Laboratory of Ultrasound Imaging and Artificial Intelligence in Medicine, Henan Provincial People’s Hospital, Zhengzhou, China; ^6^ Department of Health Management, Henan University People’s Hospital, Henan Provincial People’s Hospital, Zhengzhou University People’s Hospital, Zhengzhou, China

**Keywords:** immunotherapy, hepatocellular carcinoma, anti-PD-1 antibody, radiomics, prediction, machine learning, ultrasound

## Abstract

**Objective:**

The objective of this study is to build and verify the performance of machine learning-based ultrasomics in predicting the objective response to combination therapy involving a tyrosine kinase inhibitor (TKI) and anti-PD-1 antibody for individuals with unresectable hepatocellular carcinoma (HCC). Radiomic features can reflect the internal heterogeneity of the tumor and changes in its microenvironment. These features are closely related to pathological changes observed in histology, such as cellular necrosis and fibrosis, providing crucial non-invasive biomarkers to predict patient treatment response and prognosis.

**Methods:**

Clinical, pathological, and pre-treatment ultrasound image data of 134 patients with recurrent unresectable or advanced HCC who treated with a combination of TKI and anti-PD-1 antibody therapy at Henan Provincial People’s Hospital and the First Affiliated Hospital of Zhengzhou University between December 2019 and November 2023 were collected and retrospectively analyzed. Using stratified random sampling, patients from the two hospitals were assigned to training cohort (*n* = 93) and validation cohort (*n* = 41) at a 7:3 ratio. After preprocessing the ultrasound images, regions of interest (ROIs) were delineated. Ultrasomic features were extracted from the images for dimensionality reduction and feature selection. By utilizing the extreme gradient boosting (XGBoost) algorithm, three models were developed: a clinical model, an ultrasomic model, and a combined model. By analyzing the area under the receiver operating characteristic (ROC) curve (AUC), specificity, sensitivity, and accuracy, the predicted performance of the models was evaluated. In addition, we identified the optimal cutoff for the radiomic score using the Youden index and applied it to stratify patients. The Kaplan-Meier (KM) survival curves were used to examine differences in progression-free survival (PFS) between the two groups.

**Results:**

Twenty ultrasomic features were selected for the construction of the ultrasomic model. The AUC of the ultrasomic model for the training cohort and validation cohort were 0.999 (95%CI: 0.997-1.000) and 0.828 (95%CI: 0.690-0.966), which compared significant favorably to those of the clinical model [AUC = 0.876 (95%CI: 0.815-0.936) for the training cohort, 0.766 (95%CI: 0.597-0.935) for the validation cohort]. Compared to the ultrasomic model, the combined model demonstrated comparable performance within the training cohort (AUC = 0.977, 95%CI: 0.957-0.998) but higher performance in the validation cohort (AUC = 0.881, 95%CI: 0.758-1.000). However, there was no statistically significant difference (*p* > 0.05). Furthermore, ultrasomic features were associated with PFS, which was significantly different between patients with radiomic scores (Rad-score) greater than 0.057 and those with Rad-score less than 0.057 in both the training (HR = 0.488, 95% CI: 0.299-0.796, *p* = 0.003) and validation cohorts (HR = 0.451, 95% CI: 0.229-0.887, *p* = 0.02).

**Conclusion:**

The ultrasomic features demonstrates excellent performance in accurately predicting the objective response to TKI in combination with anti-PD-1 antibody immunotherapy among patients with unresectable or advanced HCC.

## 1 Introduction

As the sixth most frequently occurring cancer worldwide, liver cancer is the third most leading cause of cancer-related death (1, 2). The global healthcare burden posed by liver cancer is still on the rise (3). By 2040, the global annual death toll from liver cancer is expected to exceed 1.3 million people (4). Hepatocellular carcinoma (HCC) constitutes more than 80% of liver cancer cases, making it the predominant type (5). Life expectancy after HCC diagnosis is lower than that of many other cancers. Delayed diagnosis, restricted treatment choices, and absence of predictors for response to antineoplastic agents are the major reasons for poor HCC outcomes (6, 7). Most patients with HCC receive their diagnosis when their illness is either intermediate or advanced. For individuals diagnosed with unresectable or advanced HCC, palliative treatment is the only option available (8), such as molecular targeted therapy, immunotherapy or chemotherapy. As an innovative and successful therapeutic approach, tumor immunotherapy has a promising future for the management of advanced or unresectable HCC (9). HCC is regarded as an immunogenic tumor. The liver expresses immunological checkpoint molecules, including programmed cell death ligand 1 (PD-L1), programmed cell death 1 (PD-1), and cytotoxic T lymphocyte-associated protein 4 (CTLA4). Immune checkpoint inhibitors (ICIs) can lift immune system suppression by blocking these immune checkpoints (10) and thereby enhance antitumor functions (11, 12).In terms of ICI-based immunotherapy, anti-PD-1 antibodies have markedly enhanced the prognosis for patients with advanced HCC, the objective response rate (ORR) reached 17%-20% with certain patients achieving complete response (13, 14). However, studies have demonstrated that ICI monotherapy shows limited effectiveness, benefiting just a tiny subset of patients. Recent research has demonstrated that combination therapy with an ICI plus a targeted agent results in a higher ORR and holds more promising application prospects (15). In fact, the Food and Drug Administration has approved this combination therapy for use as the first-line therapy for metastatic or unresectable HCC, marking the beginning of the immunotherapy era in liver cancer (16). Tyrosine kinase inhibitor (TKI) is a class of anti-angiogenic targeted agents that effectively block tyrosine kinase activity and inhibit cell signal transduction, thereby inhibiting tumor cell growth and proliferation. TKI, including sorafenib, lenvatinib, and regorafenib, approved as first-line therapy for advanced HCC (17). It is also one of the most successful partners in combination with anti-PD-1 antibodies to date (18). In addition to the known antiangiogenic effects of TKI on tumor cells, these agents also exhibit synergistic antitumor effects through multiple mechanisms, the theoretical foundation for their combination with ICI is their activity in immune regulation (19), and their high efficiency can be utilized to enhance the anticancer effect of ICI. ICI plus TKI combination therapy has been shown to result in higher ORR in liver cancer patients compared with TKI alone (≥ 30%) (20). This combination therapy, such as lenvatinib plus anti-PD-1 antibody, achieved an ORR of up to 46% in early-phase clinical trials (15), which was significantly higher than that of immunotherapy alone or targeted therapy alone. Therefore, combination therapies with anti-PD-1 antibody and TKI are widely favored in clinical practice (21). Despite the high ORR of this combination therapy, due to the 50% occurrence of severe (grade 3-4) adverse events (22), probability of adverse events is high and severe, and it benefits only a select group of patients. It is therefore crucial in clinical practice to identify patients who will benefit or are more susceptible to serious adverse reactions prior to initiation of combination therapy. Nevertheless, no reliable biomarkers can be accurately predicted response to combined targeted therapy and immunotherapy (23). Therefore, it is particularly important to explore the potential of imaging as a non-invasive monitoring tool.

The impact of immunotherapy on the tumor microenvironment is profound. Research has shown that immunotherapy can alter the cellular composition within tumors, including immune cell infiltration and the activation of tumor-associated fibroblasts, which may lead to changes in tumor vascularity and structural remodeling (24). These changes may be reflected in radiomic features, such as tumor texture, density, and shape, providing crucial non-invasive biomarkers for assessing treatment response.

Radiomics is a developing technology that captures high-throughput radiomic features from radiological images with high sensitivity, which reflect tumor microenvironment and heterogeneity (25–27). These radiomic features are closely associated with tumor biological behavior and may provide important insights for evaluating the response to immunotherapy. Multiple studies have shown that superior predictive ability of radiomics for immunotherapy response in breast cancer, lung cancer, and renal cancer. However, most of these studies utilized computed tomography (CT) or magnetic resonance imaging (MRI) (28–32). For example, studies based on MRI and contrast-enhanced CT showed that radiomic models have demonstrated precise prediction capabilities for responses to TKI, TKI plus anti-PD-1 antibody, and anti-PD-1 antibodies for advanced HCC patients (33–35), thus preliminarily supporting its favorable predictive performance. However, both MRI and contrast-enhanced CT imaging are limited in application to specific populations. For example, MRI is not suitable for patients with metal implants (e.g., dentures or cardiac stents) or claustrophobia, while contrast-enhanced CT is not suitable for patients with allergy to contrast agents. Ultrasound is a conventional imaging modality widely used in clinical diagnosis and treatment (36). Ultrasound has several advantages over MRI and CT, including shorter examination and wait time, no radiation, and lower costs. Ultrasomics is a branch of radiomics that has shown favorable performance in the diagnosis (37), pathological grading (38, 39), immunohistochemistry (40, 41) and prognostic prediction (42, 43) of HCC. However, the predictive performance of ultrasomics in assessing the response to TKI combined anti-PD-1 antibody therapy for advanced HCC patients has been rarely studied, this study intends to explore this, developing and validating a machine learning-based ultrasomic model to predict the objective response of patients with unresectable HCC to TKI combined with PD-1 antibody therapy, in order to provide novel therapeutic options for who were intermediate- to advanced stage liver cancer patients.

## 2 Materials and methods

### 2.1 Study design and subjects

This was a retrospective, two-center study. Clinical, pathological and ultrasound information were collected from 1,128 patients with intermediate/advanced or recurrent unresectable HCC treated with a TKI combination with anti-PD-1 therapy at Henan Provincial People’s Hospital (institution I) and the First Affiliated Hospital of Zhengzhou University (institution II) between December 2019 and November 2023. These data included: (1) Demographic and clinical characteristics: gender, age, cirrhosis, Child-Pugh classification, and treatment plan; (2) Laboratory indices: Hepatitis B surface antigen (HbsAg)/hepatitis C virus antibody (HCV-Ab), carcinoembryonic antigen (CEA), alpha-fetoprotein (AFP), carbohydrate antigen 19-9 (CA19-9), total bilirubin (TBIL), alanine aminotransferase (ALT), glutamyl transpeptidase (GGT), aspartate aminotransferase (AST), prothrombin time (PT) level, and D-dimer; (3) Histopathological features: Barcelona Clinic Liver Cancer (BCLC) staging (44), maximum tumor diameter, metastasis, and tumor emboli; (4) Ultrasound image acquisition: Ultrasound images of the lesion(s) within two weeks before the first treatment; (5) Follow-up information: Prognostic indicators such asprogression-free survival (PFS).

**Figure 1:**
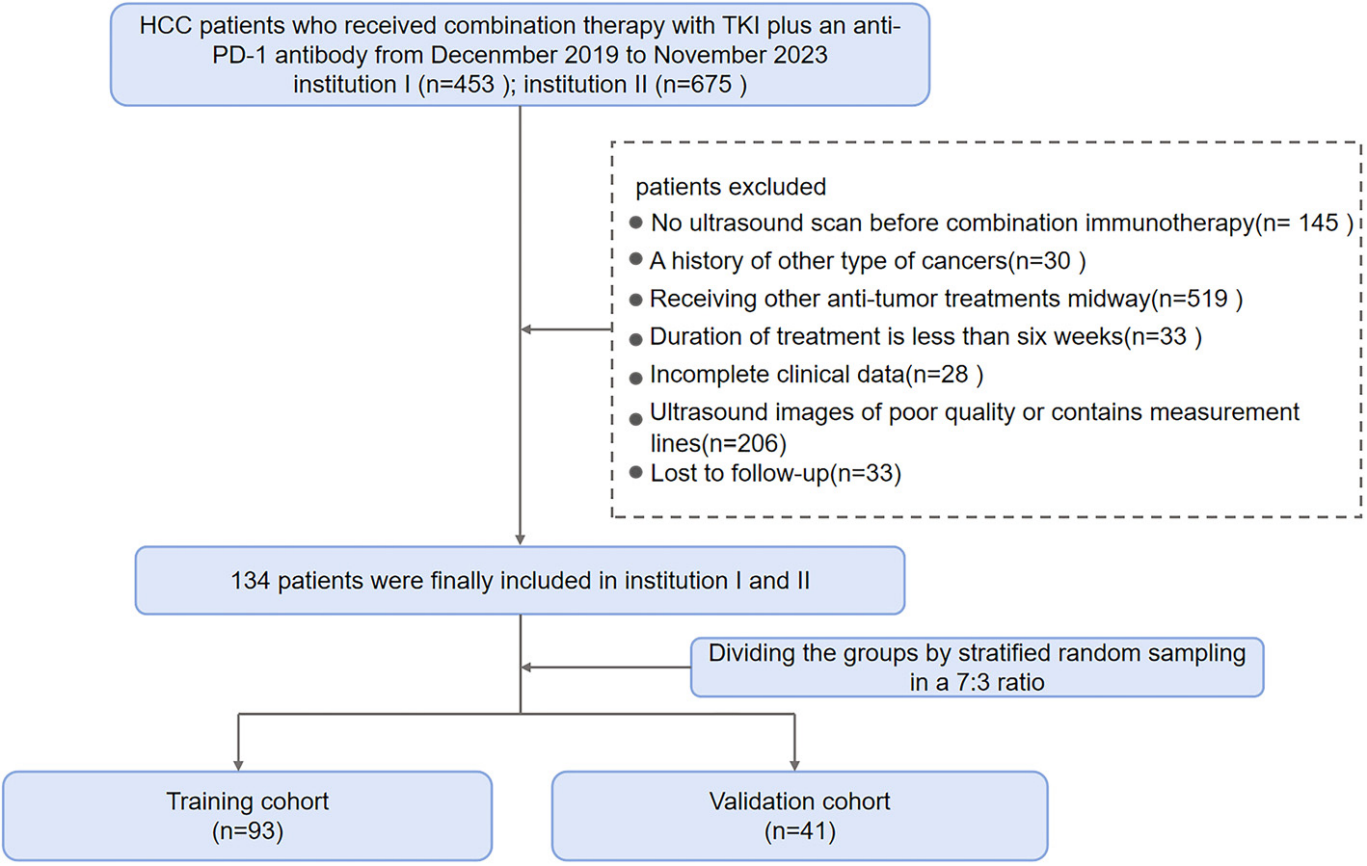
Flow diagram illustrating patient inclusion and exclusion criteria.

Criteria for screening patients were as follows. Main inclusion criteria: (1) ≥ 18 years old, diagnosed with HCC through two imaging modalities or a biopsy; (2) Regular TKI plus anti-PD-1 treatment for at least 2 cycles; (3) Liver ultrasound examination within 2 weeks before treatment and complete ultrasound imaging data; (4) No prior history of cancer aside from HCC; (5) Measurable lesions identified in accordance with Response Evaluation Criteria in Solid Tumors (RECIST) version 1.1; and (6) At least 2 months between the commencement of combination therapy and any prior treatment (e.g., TACE, radiofrequency ablation, liver resection). Exclusion criteria: (1) Local treatment during the follow-up period; (2) Incomplete clinical data; (3) Incomplete, unclear or obstructed ultrasound images; (4) Loss to follow-up. A final total of 134 eligible HCC patients were included from the two centers and randomly allocated to the training cohort (n = 93) and validation cohort (n = 41) at a 7:3 ratio using stratified random sampling. **Figure 1** illustrates the patient enrollment process.

The research was approved by both healthcare facilities’ ethical review boards and followed the standards of the Declaration of Helsinki. All patients signed a consent form for anti-PD-1 treatment before each treatment.

### 2.2 Parameter definition and subsequent strategy

All patients received oral TKIs (including lenvatinib, apatinib, sorafenib, and regorafenib) once daily at a dose determined based on the patient’s weight. Anti-PD-1 antibody was given intravenously once every 21-day treatment cycle, starting on day 1. Toripalimab was administered intravenously at 3 mg/kg body weight, and sintilimab, camrelizumab, and tislelizumab were all administered intravenously at a fixed dose of 200 mg. Treatment response was assessed every two treatment cycles, and objective tumor response was assessed at least once following the commencement of combination therapy.

PFS has been defined as the time between therapy beginning and first progression or mortality for any cause. The observation cutoff date was February 29, 2024.

Tumor response, the outcome of the study, was assessed by a senior oncologist and a junior radiologist according to RECIST V.1.1 (45). The assessment period was defined as 4–12 weeks after treatment (33). Objective response has been measured by the changes in tumor size on imaging, along with considerations of clinical factors such as the development of new lesions.

The major outcome of the work was the response to combination therapy, evaluated for complete response (CR), partial response (PR), stable disease (SD), and progressive disease (PD) based on RECIST V1.1. In this study, patients achieving PR and CR were categorized as ‘objective responders’, while those in SD and PD served as ‘non-objective responders’ (28).

### 2.3 Image acquisition, preprocessing and ROI segmentation

Patients underwent an abdominal ultrasound examination in the supine position under fasting conditions within 2 weeks prior to the first treatment. Abdominal ultrasound images were obtained using a convex array transducer at a frequency of 2.5-7.5 MHz. The equipment models were GE Logiq E20, Philips EPIQ 7, SIEMENS and other types. Clear original ultrasound images of tumor lesions were selected and exported in DICOM format. Ultrasound images were preprocessed by experienced researchers to ensure that the extracted features are comparable and ensure consistency across various equipment and operators. Specifically, a B-spline curve was used to resample images of various voxel sizes to a pixel size of 1 mm x 1 mm. Additionally, gray level discretization was performed by setting a fixed bin width of 25 in the histogram.

**Figure 2:**
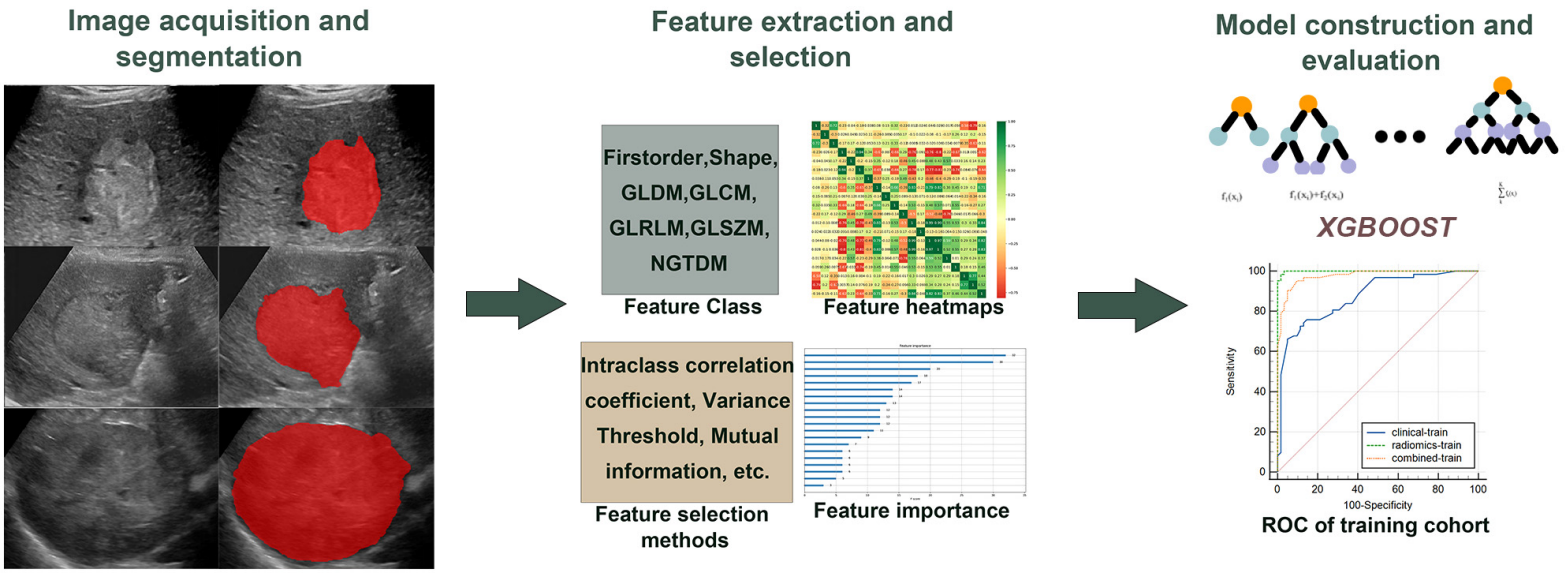
Study flow chart. The study procedures encompassed image acquisition, ROI segmentation, extraction and selection of features, and model construction and evaluation.

Images were segmented into regions of interest (ROI) by two sonographers. First, under the guidance of a senior sonographer (Sonographer 1), a junior sonographer (Sonographer 2) manually outlined the regions of interest (ROI) along the edges of tumor lesions on each ultrasound image using ITK-SNAP 3.8 (http://www.itksnap.org). Both Sonographer 1 and Sonographer 2 received standardized training during the ROI segmentation process to ensure consistency and reliability in the segmentation outcomes. To evaluate how consistently features were identified between observers, Sonographer 1 randomly selected 50 ultrasound images for re-segmentation. Both sonographers were kept unaware of the clinical data and treatment outcomes for entirety patients. Lesion segmentation and subsequent procedures are shown in **Figure 2**.

### 2.4 Extraction and screening of ultrasomic features

Ultrasomic features were extracted using the Python package Pyradiomics v.2.1.2. The original images were processed by 14 filters to generate the derived images. Seven categories of features obtained from the original and derived images: first-order, shape features, gray-level size zone matrix (GLSZM), gray-level co-occurrence matrix (GLCM), gray-level dependency matrix (GLDM), gray-level run length matrix (GLRLM), and neighboring gray tone difference matrix (NGTDM). Details regarding feature extraction and applied filters are summarized in **Supplementary Material 1**.

To make the distribution of features uniform, the ultrasomic features were standardized through Z-score for further analysis. However, a vast array of high-dimensional features extracted could reduce computational efficiency and elevate the risk of overfitting. Consequently, dimensionality reduction was performed to identify valuable features. First, the reproducibility (inter-observer agreement) of features extracted from ROI was measured by calculating the intra-class correlation coefficient (ICC). ICC > 0.8 demonstrated good reliability (46), and the analysis comprised characteristics with ICC > 0.8. Second, features with a variance of 0 were excluded using the variance filtering method. Then, any relationship (linear or nonlinear) between each feature and the classification label was captured by mutual information method, and features with maximum information coefficient (MIC) of 0 were excluded. Last, further feature dimensionality reduction was accomplished using the embedding method combined with extreme gradient boosting (XGBoost) to select the most valuable features.

### 2.5 Model construction and evaluation

Three prediction models were built using the XGBoost algorithm, namely the ultrasomic model, clinical model and combined model. XGBoost is an efficient machine learning (ML) algorithm that integrates optimized regularization techniques and parallelization strategies to improve the generalizability, training efficiency, and thus robustness of the model (47).

Initially, the ultrasomic model was developed using the ultrasomic features identified through the above steps from the lesion ROIs in the ultrasound images of the patients. Second, the clinical model was developed based on clinical characteristics of all patients, such as sex and age. Last, the above clinical and ultrasomic features were integrated to construct a combined model.

The predictive efficacy concerning the three models was assessed within training and validation cohorts based on metrics such as area under the receiver operating characteristic curve (AUC) with a 95% confidence interval, sensitivity, accuracy, and specificity.

### 2.6 Statistical analysis and survival analysis

Statistical analyses were performed using R (V.4.4.0, http://www.R-project.org) and SPSS V.25.0 (IBM SPSS V.25.0, Chicago, USA). Continuous variables were expressed as mean ± standard deviation or median [inter quartile range (IQR)], while the categorical variables were expressed as counts and percentages. Data comparisons for the training and validation cohorts were performed using the t-test for normally distributed variables or the Mann-Whitney test for non-normally distributed variables. The performance of each model was assessed using the ROC curve and AUC, and the AUCs of the models were compared using the Delong test. A *p* < 0.05 was regarded as statistically significant.

We used the radiomics-based Rad-score to forecast the prognosis of patients with HCC. The optimal cutoff was calculated using the Youden’s index. PFS was determined by the Kaplan-Meier (KM) survival curves, and the log-rank test was used to get the *p*-value.

## 3 Results

### 3.1 Baseline clinical characteristics

In this study, we screened a total of 1,128 patients with hepatocellular carcinoma who received treatment with a combination of TKI and anti-PD-1 antibody. Eventually, an overall number of 134 eligible HCC patients from two healthcare institutions were included in the work and allocated into the training cohort (*n* = 93) and validation cohort (*n* = 41). In this study, males and females accounted for 83.58% (112/134) and 16.42% (22/134) of the total patient population, respectively. The percentages of responders and non-responders to treatment were 33.58% (45/134) and 66.42% (89/134), respectively. PR was achieved in 45 patients (33.58%), SD in 62 patients (46.27%), and PD in 27 patients (20.15%). Baseline demographic and disease characteristics had no significant differences between training and validation cohorts (**Table 1**). ORR was 33.33% (31/93) in the training cohort and 34.15% (14/41) in the validation cohort (*p* = 0.927).

**Table 1:** Baseline characteristics of unresectable HCC patients receiving combined immunotherapy in the training and validation cohorts.

Variables	All patients (*n* =134)	Training cohort (*n* =93)	Validation cohort (*n* =41)	*P* value
Gender				0.521
Female	22 (16.42%)	14 (15.10%)	8 (19.50%)	
Male	112 (83.58%)	79 (84.90%)	33 (80.50%)	
Age (year), mean ± SD	59.74 ± 10.10	58.49 ± 10.25	62.56 ± 9.25	0.031
HbsAg/HCV-Ab				0.418
positive	110 (82.09%)	78 (83.90%)	32 (78.00%)	
negative	24 (17.91%)	15 (16.10%)	9 (22.00%)	
Child-Pugh				0.609
A	57 (42.54%)	41 (44.10%)	16 (39.00%)	
B	75 (55.97%)	51 (54.80%)	24 (58.50%)	
C	2 (1.49%)	1 (1.10%)	1 (2.40%)	
Tumor response				0.927
Yes	45 (33.58%)	31 (33.33%)	14 (34.15%)	
No	89 (66.42%)	62 (66.78%)	27 (65.85%)	
AFP (ng/ml)	148.63 (19.65-1040.25)	150.65 (12.02-1118.00)	132.00 (32.70-1033.50)	0.938
CEA (ng/ml)	2.25 (1.59-3.25)	2.26 (1.59-3.21)	2.10 (1.59-4.09)	0.800
CA199 (U/ml)	22.315 (10.43-51.95)	22.33 (12.85-52.40)	17.60 (8.63-51.91)	0.343
AST, >40 (U/L)	65 (48.51%)	45 (48.40%)	20 (48.80%)	0.967
ALT, >40 (U/L)	85 (63.43%)	59 (63.40%)	26 (63.40%)	0.998
GGT, >58 (U/L)	106 (79.10%)	73 (78.50%)	33 (80.50%)	0.794
ALP, >130 (U/L)	74 (55.22%)	53 (57.00%)	21 (51.20%)	0.536
TBIL, >25 (μmol/L)	29 (21.64%)	23 (24.70%)	6 (14.60%)	0.191
D_dimer,>0.3 (mg/L)	105 (78.36%)	72 (77.40%)	33 (80.50%)	0.691
PT,>13.6 (s)	33 (24.63%)	22 (23.70%)	11 (26.80%)	0.694
Liver cirrhosis	106 (79.10%)	76 (81.70%)	30 (73.20%)	0.262
Long-diameter,≥50 (mm)	78 (58.21%)	54 (58.06%)	24 (58.54%)	0.889
Tumor number				0.642
Single	42 (31.34%)	28 (30.10%)	14 (34.10%)	
Multiple	92 (68.66%)	65 (69.90%)	27 (65.90%)	
BCLC stage				0.896
A	1 (0.75%)	1 (1.10%)	–	
B	58 (43.28%)	41 (44.10%)	17 (41.50%)	
C	75 (55.97%)	51 (54.80%)	24 (58.50%)	
cancer_embolus	64 (47.76%)	44 (47.30%)	20 (48.80%)	0.875
Tumor responses				0.661
PR	45 (33.58%)	31 (33.33%)	14 (34.15%)	
SD	62 (46.27%)	45 (48.39%)	17 (41.46%)	
PD	27 (20.15%)	17 (18.28%)	10 (24.39%)	

Unless otherwise specified, data for n (%) or median (IQR). IQR, interquartile range; AFP, alpha-fetoprotein; CEA, carcinoembryonic antigen; CA19-9, carbohydrate antigen 19-9; ALP, alkaline phosphatase; ALT, alanine aminotransferase; AST, aspartate aminotransferase; GGT, glutamyl transpeptidase; HCC, hepatocellular carcinoma; PT, prothrombin time; TBIL, total bilirubin; BCLC, Barcelona Clinic Liver Cancer; PR, partial response; SD, stable disease; PD, progressive disease.

Univariate logistic regression analysis of agents used by responders and non-responders showed that different TKIs or anti-PD-1 antibodies had no significant effect on treatment response (*p* > 0.05) (**Table 2**).

**Table 2:** Univariate logistic regression analysis of the effect of different TKIs and anti-PD-1 antibodies on response in unresectable HCC.

		Univariate Analysis	
	HR	95%CI	*P* value
Anti-PD-1 Antibody			0.094
Sintilimab		Reference	
Camrelizumab	0.437	0.184-1.037	0.061
Tislelizumab	0.530	0.200-1.405	0.202
Toripalimab	4.174	0.408-42.716	0.229
TKI			0.476
lenvatinib		Reference	
Apatinib	0.916	0.334-2.511	0.864
Sorafenib	0.690	0.266-1.792	0.447
Regorafenib	1.636	0.592-4.521	0.343

HR, hazard ratio; CI, confidence interval: HCC, hepatocellular carcinoma; TKI, tyrosine kinase inhibitor.

### 3.2 Feature extraction and screening

A combined sum of 1,409 features were obtained from patients’ both original and derived ultrasound images. These features were classified as first-order (18), shape features (14), GLSZM (16), GLCM (24), GLDM (14), GLRLM (16), and NGTDM (5). First, features with an ICC < 0.8 were not considered, retaining 1054 features that were considered robust. Second, 16 features with a variance of 0 were removed using the variance filtering method, and 530 features with a MIC of 0 were excluded by the mutual information method. Last, the features were subjected to further dimensionality reduction by the embedding method combined with XGBoost. Ultimately, 20 most valuable features were obtained after screening. The importance and heat map of features are shown in **Supplementary Figures S2**, **S3** and **Supplementary Material S2**.

### 3.3 Model construction and performance comparison

**Figure 3:**
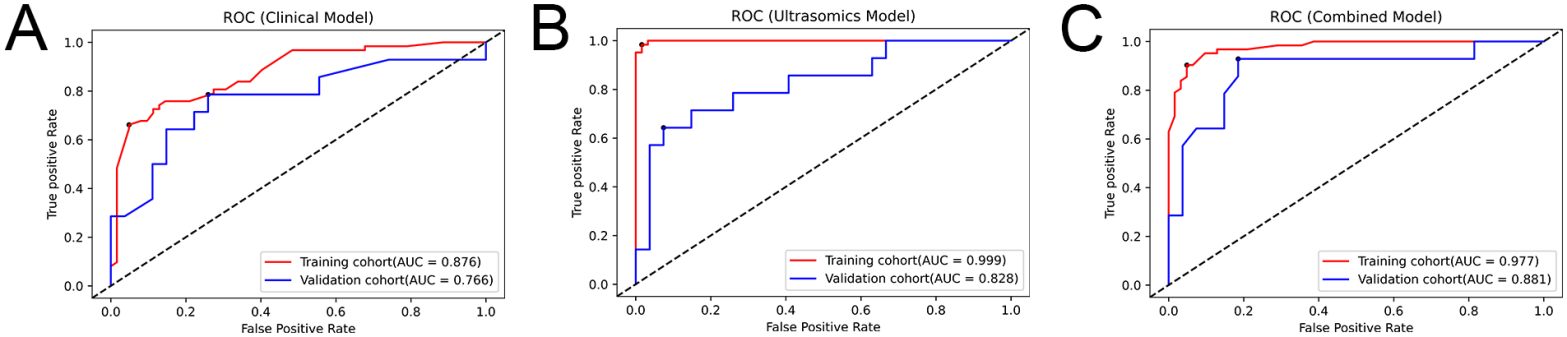
ROC curves depicting the performance of the three models in the training and validation cohorts. **(A)** Clinical model; **(B)** Ultrasomic model; **(C)** Combined model.

Three prediction models were built using XGBoost: the ultrasomic model, clinical model, and combined model. **Supplementary Material 3** contains additional information regarding the construction of the models. The results show that though all three models could predict the response to TKI combined with anti-PD-1 treatment in HCC, the AUCs of the ultrasomic model for the training cohort and validation cohort were 0.999 (95%CI: 0.997-1.000) and 0.828 (95%CI: 0.690-0.966), which were similar to those of the combined model [0.977 (95%CI: 0.957-0.998) and 0.881 (95%CI: 0.758-1.000), respectively] all higher than those of the clinical model [0.876 (95%CI: 0.815-0.936) and 0.766 (95%CI: 0.597-0.935), respectively]. When ultrasomic features were combined with clinical characteristics, the combined model did not show significant improvement in predicting response to TKI in combination with anti-PD-1 therapy compared to the ultrasomic model. **Figure 3** displays the ROC curves for all three models in both training and validation cohorts, with detailed performance evaluation presented in **Table 3**.

**Table 3:** Performance of each model in the training cohort and validation cohort.

Cohort	Model	Accuracy (%)	Sensitivity (%)	Specificity (%)	AUC (95%CI)	*P* value
Training cohort	Clinical	75.81	79.03	72.58	0.876 (0.815-0.936)	<0.0001
	Ultrasomics	98.39	98.39	98.39	0.999 (0.997-1.000)	<0.0001
	Combined	91.13	96.77	85.48	0.977 (0.957-0.998)	<0.0001
Testing cohort	Clinical	73.17	64.29	77.78	0.766 (0.597-0.935)	0.006
	Ultrasomics	73.17	71.43	74.07	0.828 (0.690-0.966)	0.001
	Combined	82.93	85.71	81.48	0.881 (0.758-1.000)	<0.0001

AUC, area under the curve; CI, confidence interval.

### 3.4 Prognostic performance of ultrasomic features

**Figure 4:**
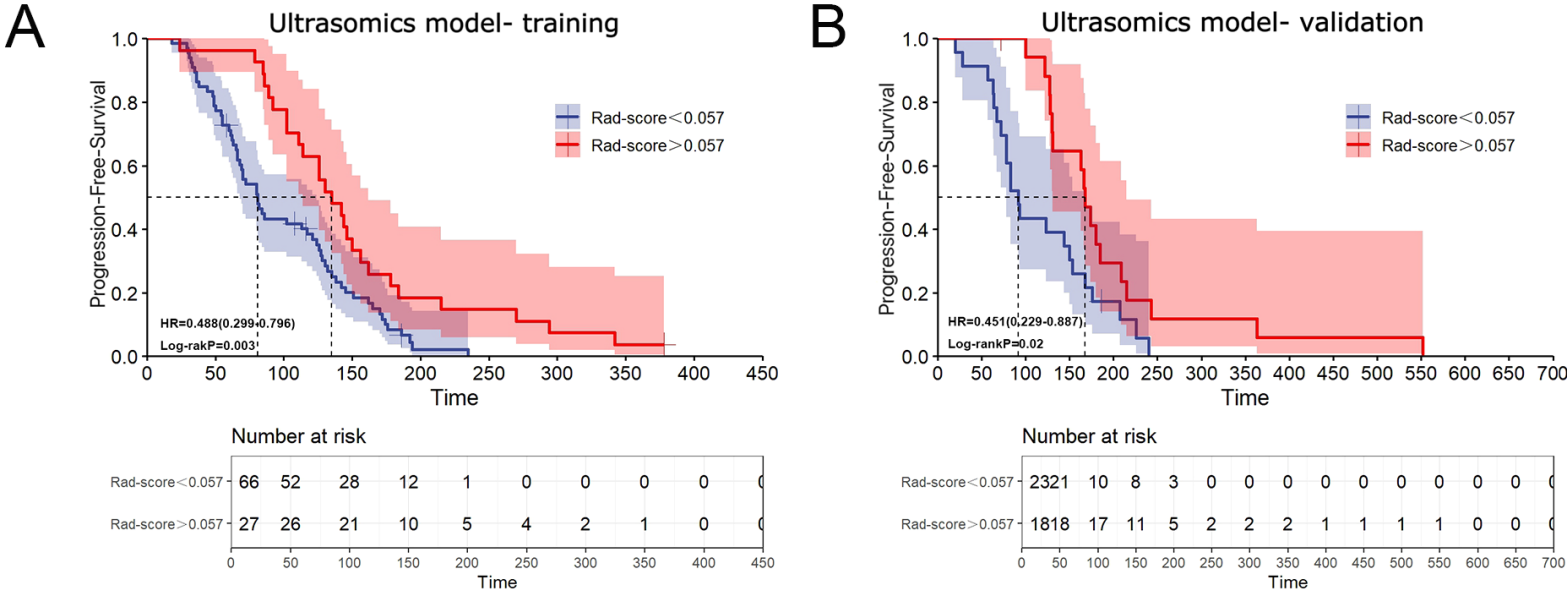
Association between ultrasomic features and treatment prognosis. **(A)** Kaplan-Meier curve illustrating the relationship between ultrasomic features and PFS in the training cohort; **(B)** Kaplan-Meier curve illustrating the relationship between ultrasomic features and PFS in the validation cohort.

Based upon an optimal Rad-score cut-off of 0.057, patients were stratified into two risk groups (**Supplementary Material 4**). Survival analysis revealed that PFS was significantly different between patients with Rad-score greater than 0.057 and those with Rad-score less than 0.057 in both the training and validation cohorts (HR = 0.488, 95% CI: 0.299-0.796, *p* = 0.003; and HR = 0.451, 95% CI: 0.229-0.887, *p* = 0.02) (**Figure 4**). The median PFS of patients with Rad-score greater than 0.057 was nearly twice as long as that of patients with score less than 0.057 (training cohort: 135 d (95%CI: 107.860-162.140) vs. 81 d (95%CI: 65.313-96.687); validation cohort: 168 d (95%CI: 153.210-182.790) vs. 92 d (95%CI: 74.392-109.608), respectively).

## 4 Discussion

Immunotherapy is a significant breakthrough in treating advanced or unresectable HCC (48, 49), bringing a new approach to tumor treatment and ushering in a new era of tumor immunotherapy (50). It has also significantly improved outcomes and provided favorable clinical benefits to HCC patients (51). However, due to the differences in immune microenvironment among patients and severe side effects, immunotherapy provides benefits to a limited subset of patients. Therefore, accurate and noninvasive identification of patients with objective responses to immunotherapy prior to treatment remains a key challenge in clinical practice (9). Previous studies have shown that some biomarkers like PD-1 and so on can predict whether a patient will respond to immunotherapy. However, these biomarkers require invasive biopsy, are costly, and may pose certain risks. They may also not indicated to patients with intermediate to advanced HCC whose tumors are unresectable or in poor physical condition. In addition, the efficacy of biomarkers to predict immunotherapy response is also less than ideal (52).

Radiomics is an advanced technique of analyzing images that thoroughly mines high-throughput information within images, uncovering radiomic features that imperceptible to unaided eye. This approach proves particularly valuable for disease diagnosis and prognosis prediction (53). Radiomic features can reflect the microstructure and biological characteristics of tumors. Studies have shown that certain features, such as GLCM and shape characteristics, have significant correlations with tumor cell density, necrotic areas, and the degree of fibrosis (54). These features can provide information regarding tumor response and pathological changes after treatment. For instance, changes in radiomic features can indicate alterations in tumor blood supply, thereby predicting improvements or deterioration in treatment outcomes (55). As a branch of radiomics, ultrasomics also plays a key role in liver cancer diagnosis and management. In our study, we developed an ultrasomic model, a clinical model, and a combined model derived from pre-treatment ultrasound images to noninvasively predict the objective response to TKI combined with anti-PD-1 therapy in unresectable HCC. Clinical factors provide background information about the patients, while radiomic features reflect the biological characteristics of tumors. The combination of both can help identify individual differences in immune therapy responses among patients. We found that the ultrasomic model and combined model displayed good prediction performance in the training and validation cohorts from two centers, and the extracted ultrasomic features were closely associated with PFS. Therefore, the ML-based ultrasomic model serves as an accurate and noninvasive tool for predicting objective response to TKI and anti-PD-1 combination therapy for HCC patients, which is essential for developing management strategies and enhancing patient outcomes. As far as we are aware, this research is the first to utilize an ultrasomic model to noninvasively predict the effectiveness of TKI plus anti-PD-1 combination therapy for HCC.

There have been several studies on the association between the radiomic features in HCC patients and immunotherapy responses, mainly based on MRI and CT imaging techniques (33–35). Xu et al. evaluated the performance of a radiomic model based on pre-treatment MRI images in predicting objective response to lenvatinib combined with anti-PD-1 antibody in advanced HCC (34). They constructed a clinicopathological model and a radiomic model based on selected clinicopathological and radiomic features, respectively. The AUCs of the two models were 0.748 (95% CI: 0.656-0.840) and 0.702 (95% CI: 0.547-0.884) in the training cohort (*n* = 124), and 0.886 (95% CI: 0.815-0.957) and 0.820 (95% CI: 0.648-0.984) in the validation cohort (*n* = 46), demonstrating that the radiomic model demonstrated superior predictive capabilities. In addition, the authors also found that the radiomic features showed correlation with overall survival (OS) and PFS. Bo et al. constructed 10 ML-based radiomic models using contrast-enhanced CT features for predicting the efficacy of lenvatinib for advanced or unresectable HCC (33). Among these 10 ML algorithms, AutoGluon demonstrated the most accurate predictive capacity in the training cohort (*n* = 74) (AUC = 0.97) and favorable performance in validation cohort (*n* = 35) (AUC = 0.93). Furthermore, using K-means clustering, two radiomic subtypes were identified, and subtype 1 was shown to be linked to longer PFS and OS in the Kaplan-Meier curves. Yuan et al. developed a radiomic nomogram using pre-treatment contrast-enhanced CT images to predict immune response to anti-PD-1 antibody in HCC patients (35). The radiomic nomogram incorporated eight radiomic features and two clinical characteristics and demonstrated an AUC of 0.894 (95% CI: 0.797–0.991) in the training cohort (*n* = 40) and 0.883 (95% CI: 0.716–0.998) in the testing cohort (*n* = 18). Wei et al. constructed 10 radiomic models developed from pre-treatment CT images to assess and predict treatment effectiveness of TKI plus anti-PD-1 therapy in HCC (*n* = 55) (56). The support vector machine (SVM) model presented superior performance, reaching an AUC of 0.933 in training set and 0.792 in testing set.

In agreement with these findings, our study demonstrated that the clinical model had a suboptimal performance in predicting the efficiency of TKI plus anti-PD-1 treatment in HCC patients, while the ultrasomic model exhibited satisfactory efficacy in both the training cohort [*n* = 93; AUC = 0.999 (95% CI: 0.997-1.000)] and validation cohort [*n* = 41; AUC = 0.828 (95% CI: 0.690-0.966)]. The combined model based on imaging and clinical data showed similar performance to ultrasomic model in the training cohort, with an AUC of 0.977(95%CI: 0.957-0.998), and increased to 0.881(95%CI: 0.758-1.000) in the validation cohort, but there was no statistically significant difference in comparison to the ultrasomic model (*p* > 0.05). Additionally, the extracted ultrasomic features correlated with PFS, demonstrating potential value in prognosis prediction. The ultrasomic model developed in the training and validation cohorts from two centers in the study had similar or even better predictive performance than the aforementioned MRI- or CT-based radiomic models. This also indicates that grayscale ultrasound images contain a significant amount of information, which shows strong potential for predicting tumor heterogeneity levels. Ultrasound is a commonly used imaging modality for liver diseases. It is more convenient, faster, and less expensive than MRI and CT, and is therefore more extensively used in clinical practice.

This work presents a few limitations, however. First, the sample size of the study is limited, and only two medical centers are included, which lacks many external validations. Insufficient sample size may lead to model instability, affecting its generalization ability. Given the recent emergence of immunotherapy and the stringent inclusion/exclusion criteria in this work, many patients were excluded as a result of concurrent use of other local treatments during the study, resulting in relatively small study cohort. As more patients undergo immunotherapy, further multicenter large-cohort studies are warranted to supplement and enhance external validation to verify and improve the generalization ability of the model. Second, this study only analyzed two-dimensional grayscale ultrasound images, while contrast-enhanced ultrasound can provide richer hemodynamic information, potentially improving the identification of tumor characteristics. We will consider incorporating radiomic features from contrast-enhanced ultrasound in future studies to further validate the effectiveness of our model. Last, treatment responses may vary among patients receiving different TKIs and anti-PD-1 antibodies. Our analysis showed that different medications had no significant effect on treatment response. Additionally, studies have reported comparable ORR among combination therapies with TKI and different anti-PD-1 antibodies. Future studies will need to be expanded to predict response to each kind.

In summary, our ultrasomic model performed well in noninvasively predicting the efficacy of TKI plus anti-PD-1 therapy in HCC, providing an important tool for non-invasively assessing the tumor microenvironment. The findings of this study may offer valuable insights for optimizing HCC treatment strategies and avoid unnecessary side effects and guiding clinicians in developing more accurate treatment plans.

## Data Availability

The original contributions presented in the study are included in the article/**Supplementary Material**. Further inquiries can be directed to the corresponding authors.
