# Supplementary material for: Machine learning-based ultrasomics for predicting response to tyrosine kinase inhibitor in combination with anti-PD-1 antibody immunotherapy in hepatocellular carcinoma: a two-center study
Source: Front Oncol. 2024 Nov 14;14:1464735. doi: 10.3389/fonc.2024.1464735 (PMC11602396; doi:10.3389/fonc.2024.1464735)
Supplement: Supplementary file 1 [file DataSheet1.doc]

Supplementary Material

# Supplementary 1: Feature extraction

In this study, feature extraction was performed using the Pyrodiomics v.2.1.2 package from the original images and derived images obtained by processing the original images through 14 filters. These derived images include: Wavelet (wavelet-LLH, wavelet-LHL, wavelet-LHH, wavelet-HLH, wavelet-HLL, wavelet-LLL, wavelet-HHL,wavelet-HHH), Square, Squareroot, Logarithm, Exponential, Gradient, LocalBinaryPattern 2D.

# Supplementary 2: Feature selection

Feature selection was progressively screened by inter-group correlation coefficient (ICC), variance filtering, mutual information and embedding methods combined with eXtreme Gradient Boosting (XGBoost). Finally, 20 ultrasomics features were selected, as shown in the following table.

| No. | Feature Name | Feature importance |
| --- | --- | --- |
| 1 | original_glszm_ZonePercentage | 13 |
| 2 | wavelet-LLH_glszm_LargeAreaLowGrayLevelEmphasis | 6 |
| 3 | wavelet-LLH_gldm_SmallDependenceLowGrayLevelEmphasis | 6 |
| 4 | wavelet-LHL_firstorder_Minimum | 30 |
| 5 | wavelet-LHL_glszm_SizeZoneNonUniformityNormalized | 9 |
| 6 | wavelet-LHH_firstorder_Minimum | 20 |
| 7 | wavelet-LHH_gldm_DependenceEntropy | 3 |
| 8 | wavelet-HLH_firstorder_90Percentile | 6 |
| 9 | wavelet-HLH_firstorder_Kurtosis | 32 |
| 10 | wavelet-HLH_firstorder_Range | 17 |
| 11 | wavelet-HLH_glrlm_RunEntropy | 6 |
| 12 | wavelet-HHL_firstorder_90Percentile | 14 |
| 13 | wavelet-HHL_firstorder_Median | 18 |
| 14 | wavelet-HHL_firstorder_RobustMeanAbsoluteDeviation | 7 |
| 15 | wavelet-HHL_firstorder_RootMeanSquared | 5 |
| 16 | wavelet-HHL_glrlm_RunLengthNonUniformityNormalized | 12 |
| 17 | wavelet-LLL_firstorder_Range | 12 |
| 18 | square_firstorder_RootMeanSquared | 14 |
| 19 | squareroot_firstorder_10Percentile | 11 |
| 20 | gradient_firstorder_10Percentile | 12 |

**3 Supplementary 3:Model construction and training**

First, for the ultrasomics model, 20 ultrasomics features were selected for model training and evaluation. For clinical models, text data is encoded to convert text data into numeric data, for example: gender: "male" is encoded as "0" and "female" is encoded as "1." Then, these numerical data (such as gender, age, hepatitis history, AFP, ALT, AST, etc.) were normalized by Z-score to eliminate the problem of different value scales caused by different units and value ranges of different clinical data. Finally, these clinical features are used for training and evaluation of clinical models. For the combined model, the coded clinical features were combined with 20 ultrasomics features, which were used to train and evaluate the model.

The XGBoost machine learning algorithm was used to train the clinical model, the ultrasomics model and the combination model respectively in the training cohort.

**4 Supplementary 4:Calculation of Radiomics score**

The Radiomics score (Rad-score) calculation formula：Rad-score =（0.06527146× original_glszm_ZonePercentage） + （0.02466563 × wavelet-LLH_glszm_LargeAreaLowGrayLevelEmphasis） + （0.05565392 × wavelet-LLH_gldm_SmallDependenceLowGrayLevelEmphasis） + （0.03212324 × wavelet-LHL_firstorder_Minimum） + （0.04139174× wavelet-LHL_glszm_SizeZoneNonUniformityNormalized） + （0.06238441 ×wavelet-LHH_firstorder_Minimum） + （0.05953695 × wavelet-LHH_gldm_DependenceEntropy） + （ 0.03566016× wavelet-HLH_firstorder_90Percentile） + （ 0.05088576 × wavelet-HLH_firstorder_Kurtosis）+（0.047642 ×wavelet-HLH_firstorder_Range）+（0.06368819×wavelet-HLH_glrlm_RunEntropy） + （ 0.03344465 ×wavelet-HHL_firstorder_90Percentile） + （0.04989335 × wavelet-HHL_firstorder_Median） + （0.03137181 ×wavelet-HHL_firstorder_RobustMeanAbsoluteDeviation） + （0.07428378× wavelet-HHL_firstorder_RootMeanSquared） + （0.06768369 ×wavelet-HHL_glrlm_RunLengthNonUniformityNormalized） + （ 0.02881851× wavelet-LLL_firstorder_Range） + （ 0.0347607× square_firstorder_RootMeanSquared） + （ 0.07923687 × squareroot_firstorder_10Percentile）+（0.06160321× gradient_firstorder_10Percentile）.

# 5 Supplementary Figures





**Supplementary Figure 1.** The feature importance graph shows the ranking of 20 important ultrasomics features.


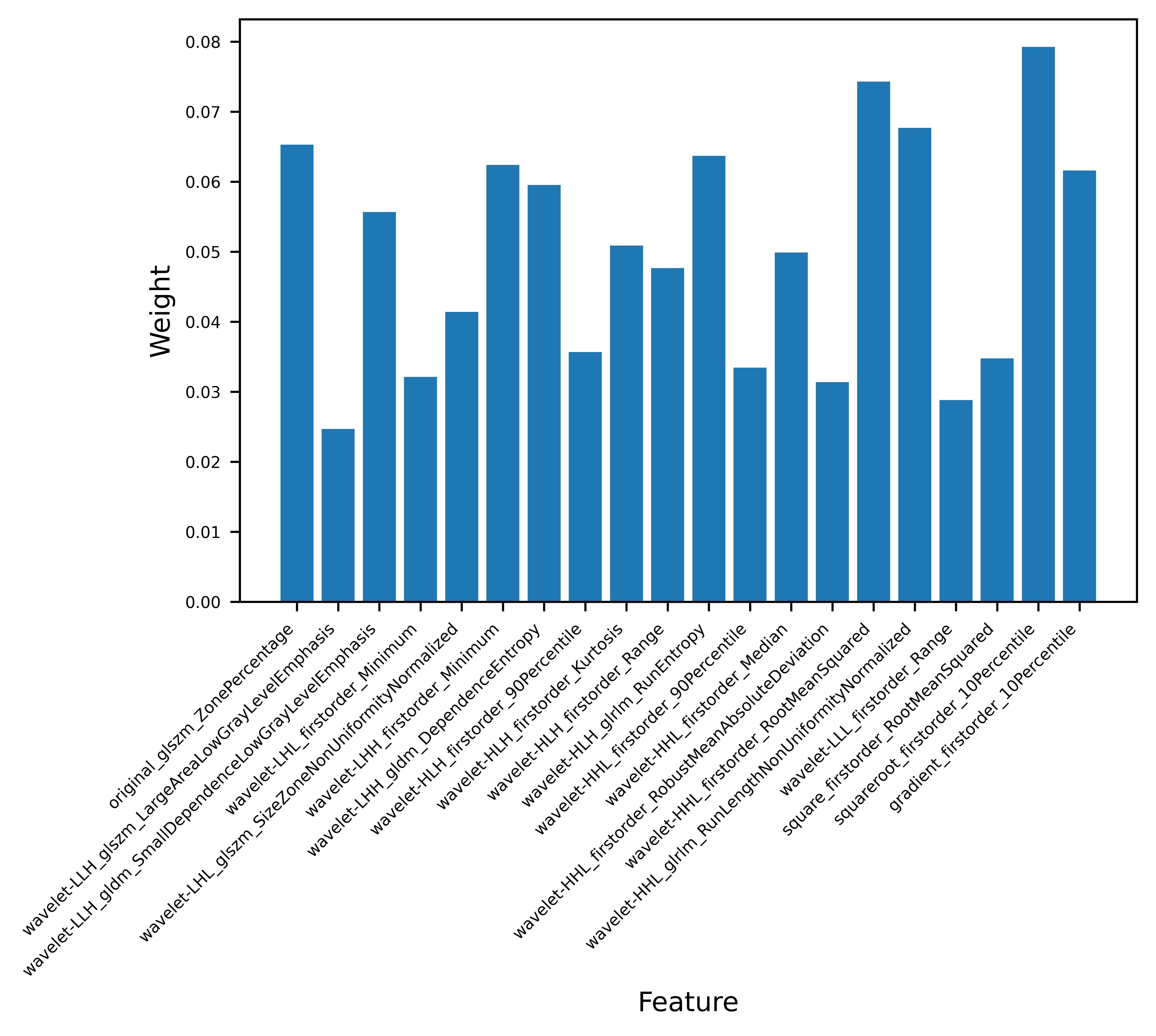


**Supplementary Figure 2.** The ultrasomics feature graph shows the coefficients corresponding to each of the 20 important ultrasomics features.





**Supplementary Figure 3.**The feature heat map shows the correlation between 20 important ultrasomics signatures extracted by color from the training cohort. The redder the color, the weaker the correlation, and conversely, the greener the color, the stronger the correlation.
